# Dilation of subglacial sediment governs incipient surge motion in glaciers with deformable beds

**DOI:** 10.1098/rspa.2020.0033

**Published:** 2020-06-03

**Authors:** B. M. Minchew, C. R. Meyer

**Affiliations:** 1 Department of Earth, Atmospheric and Planetary Sciences, Massachusetts Institute of Technology, Cambridge, MA, USA; 2 Thayer School of Engineering, Dartmouth College, Hanover, NH, USA

**Keywords:** glacier surges, glacier dynamics, granular mechanics

## Abstract

Glacier surges are quasi-periodic episodes of rapid ice flow that arise from increases in slip rate at the ice–bed interface. The mechanisms that trigger and sustain surges are not well understood. Here, we develop a new model of incipient surge motion for glaciers underlain by sediments to explore how surges may arise from slip instabilities within a thin layer of saturated, deforming subglacial till. Our model represents the evolution of internal friction, porosity and pore water pressure within the till as functions of the rate and history of shear deformation, and couples the till mechanics to a simple ice-flow model. Changes in pore water pressure govern incipient surge motion, with less permeable till facilitating surging because dilation-driven reductions in pore water pressure slow the rate at which till tends towards a new steady state, thereby allowing time for the glacier to thin dynamically. The reduction of overburden (and thus effective) pressure at the bed caused by dynamic thinning of the glacier sustains surge acceleration in our model. The need for changes in both the hydromechanical properties of the till and the thickness of the glacier creates restrictive conditions for surge motion that are consistent with the rarity of surge-type glaciers and their geographical clustering.

## 1. Introduction

Surges are enigmatic characteristics of glacier flow. Broadly speaking, glacier surges are subannual to multi-annual periods of relatively rapid flow that occur quasi-periodically, with quiescent periods between surges ranging from several years to centuries [1,2]. Flow speeds during a surge can reach 5–100 times typical quiescent-phase velocities because of commensurate increases in the rate of slip at the ice–bed interface, hereafter called the basal slip rate. Accelerated basal slip rates are facilitated by changes in the mechanical, thermal and hydrological properties of the bed, which may work independently or in concert to initiate, sustain and arrest glacier surges [2–10].

Surges are known to occur in only about 1% of glaciers worldwide [11,12]. Known surge-type glaciers are clustered in a handful of globally dispersed geographical regions, share some comparable geological factors and can inhabit a variety of climates [1,12,13]. A common feature identified in some surge-type glaciers is the presence of mechanically weak beds consisting of thick layers of water-saturated, deformable sediment and erodible sedimentary or volcanic rock [14–19]. This commonality suggests that the mechanics of deformable glacier beds play an important role in initiating and sustaining glacier surges. However, the fact that not every glacier underlain by sediments surges indicates that the existence of a deformable bed is not a sufficient condition for surging [16]. Despite the prevalence of till, many existing surge models ignore till mechanics and often focus on the hydrological and thermal states [10,20].

Many existing models of glacier surges rely on an evolving subglacial hydrological system, which can influence water pressure and thereby drag at the bed [3,10,21]. One such model posits that incipient surge motion arises from a switch in the subglacial hydrological system from a relatively efficient channelized system to an inefficient distributed, or linked cavity, system [3,21], though recent work suggests that a distributed hydrological system primes but does not trigger surges [10]. Throughout the surge phase, the basal hydrological system probably remains relatively inefficient, facilitating rapid basal slip due to lubrication from high basal water pressures, until re-establishment of an efficient channelized system reduces basal water pressure and terminates the surge [10,21–24]. Given a supply of water to the bed, this theory has the potential to explain rapid surge motion and coincident increases in basal water pressure, at least in glaciers with rigid beds [21]. Indeed, observations of a subglacial flood that occurred during, but did not initiate, a surge suggest that the basal hydrological system was likely to be inefficient during the surge and became channelized just prior to surge termination [22,25]. However, surges have been observed to begin in late autumn or winter, when surface meltwater supplies are limited [21,23,26–29]. As noted by Kamb [3], often credited with introducing hydrological switching as an incipient surge mechanism, surge onset in the absence of surface meltwater flux may require an incipient surge mechanism beyond a switch from an efficient to an inefficient basal hydrological system. Furthermore, observations of numerous surge-type glaciers in Iceland show that jökulhaups, or subglacial floods, do not cause surges despite massive, rapid increases in basal water flux that characterize jökulhaups [15], and it remains unclear if hydrological models derived under the assumption of rigid, impermeable beds are applicable to glaciers with till-covered beds. In any case, hydrological models have not explained the spatial distribution of surge-type glaciers and it seems unlikely that such models can explain why many surge-type glaciers reside on deformable beds. So while the connection between surging and subglacial hydrology may be robust, the causal link between the efficiency of the basal hydrological system and surge motion remains unclear.

Another model of glacier surges, first advocated by Robin [30], contends that sediment underlying a polythermal glacier may freeze during the quiescent phase, strengthening the bed, similar to binge–purge models for Heinrich events [31–33]. As ice collects in an upstream reservoir, the thickening ice increases the overburden pressure at the bed, resulting in a corresponding decrease in the melting temperature of ice that can cause the bed to thaw and, subsequently, weaken. Warm, weakened beds facilitate basal slip, resulting in frictional heating that melts basal ice. Melted ice further lubricates the bed, leading to enhanced basal slip and more heating, thereby driving a positive thermal feedback loop [5,34,35]. Because thermal control of glacier sliding requires ice to freeze to the bed, it cannot explain surging in temperate glaciers, in which the ice is at the melting temperature and is unable to freeze to the bed. Recent observational work shows that at least some surges in polythermal glaciers initiate in temperate zones, suggesting further limitations on the applicability of thermal instability to incipient surge motion [36,37] and indicating that thermal instability is not a universal surge mechanism [34].

The prevalence of till layers beneath surge-type glaciers suggests that changes in the mechanical properties of till caused by dilation and variable pore water pressure are a promising complement to existing models of incipient surge mechanisms [10,38]. It would be difficult to overstate the complexity of granular mechanics in subglacial till [39], which is especially pronounced where the till contains coarse clasts, where ice at the ice–bed interface is laden with debris [40–42], where the ice slides over the ice–till interface [41,43,44], where clasts frozen into the ice can plough through the till [45], and where the till is mobilized during surging [46]. Even within a relatively simple layer of near-homogeneous sediment, we may expect multiple mechanisms to contribute to till deformation at any given time, including grain boundary sliding, granular flow from comminution and grain rolling, and compaction and dilation caused by shearing [47,48]. Developing models that capture all of these mechanisms is an active area of research, and we know of no current models that account for all mechanisms in a manner that satisfyingly elucidates the underlying physics. Despite these challenges, notable surge models for glaciers with deformable beds have been proposed by other authors. Truffer *et al.* [14,49] inferred till mobilization as a surge mechanism from direct observations of till deformation beneath a surge-type glacier in Alaska. Woodward *et al.* [17] proposed a conceptual model based on ice-penetrating radar surveys of a surge-type glacier in Svalbard that indicated imbricate thrust faulting. And Clarke [39] developed a physical framework for subglacial till based in part on critical state soil mechanics and an assumed viscoplastic rheology for saturated subglacial till.

Motivated in part by these models for surging in glaciers with deformable beds, we present a new physical model that leverages the mechanical properties of granular materials to help explain incipient surge motion in the absence of meltwater flux, frozen beds and frictional heating. Our model is informed by studies of soil mechanics [50], landslides [51], and earthquake nucleation and slow-slip events on tectonic faults containing water-saturated gouge [52,53]. Gouge and glacial till are mechanistically comparable materials in that both derive their strength from a fine-grained matrix [39] and, in the cases of fault breccia and till, may feature coarse clasts [54]. Regardless of the presence of coarse clasts, the load is carried by the fine-grained matrix. Laboratory experiments on fault gouge and till indicate that these materials have elastic–plastic rheologies with yield stresses defined by the normal effective stress (the difference between overburden and pore fluid pressure) and the tendency of the till to undergo internal frictional slip along grain boundaries [48,55–63]. Shear strength is a function of the rate of shearing within the till (hereafter ‘basal slip rate’ refers to the speed of the till layer in contact with the glacier) and the shear history of the till. Accounting for shear history is important because shearing can cause either dilation or compaction of granular materials, depending on the state of consolidation in the material [50]. Dilation has been identified through theory and observation as an important component controlling basal slip rates for glaciers in Svalbard and Alaska, ice caps in Iceland and ice streams in Antarctica [17,49,57,60,64–67], and here we seek to better understand the role of till compaction and dilation in incipient surge motion by developing a simple model that captures the relevant physical processes.

## 2. Model derivation

**Figure 1.**
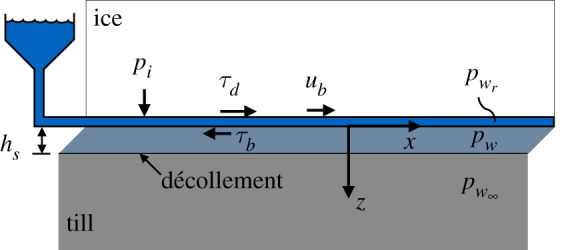
Model schematic showing a zoomed in view of the base of the idealized glacier with important parameters labelled. (Online version in colour.)

Consider a glacier with length ℓ, thickness *h* and constant width 2*w*, where *h* ≪ *w* ≪ ℓ. (Note that all variables are defined in electronic supplementary material, table 1.) Let us define a coordinate system oriented such that *x* is along flow, *y* is across flow in a right-handed configuration and *z* is downward along the gravity vector (figure 1). Assume that ice thickness is time dependent, varies along flow and is constant across flow, such that *h* = *h*(*x*, *t*).

Water-saturated till underlies the glacier. We divide the till into two layers separated by a décollement: the top layer is deformable with thickness *h*_*s*_ and pore water pressure *p*_*w*_, while the lower layer is a stationary, non-deforming half-space with pore water pressure $p_{w_{\infty }}$. Aside from strain rate, pore water pressure and otherwise stated properties, all physical properties of the till are assumed to be the same in both layers. Our idealized glacier has a subglacial hydrological system that, like any glacier, evolves as a result of changes in meltwater flux and basal slip rate [68–70]. Here, we assume that both the state of the hydrological system and the basal water flux are accounted for in $p_{w_{r}}$, the water pressure within the hydrological system, depicted as a reservoir in the system diagram (figure 1).

We assume that basal slip is due entirely to deformation of the upper till layer and there is no sliding between the ice and uppermost till layer. As a result, $p_{w_{r}}$ only influences ice flow through its influence on *p*_*w*_. We make this simplifying assumption in spite of the fact that $p_{w_{r}}$ may cause sliding of the ice relative to the bed [69,71–74] because our focus is on how the mechanical properties of till might induce surging in the absence of meltwater flux to the bed. This assumption of nearly constant $p_{w_{r}}$ is merely conceptual and is not a necessary condition in the subsequent derivation because time-varying $p_{w_{r}}$ is accounted for in the model. Indeed, in future work, subglacial hydrological models could be readily bolted onto the model presented here. For simplicity, we ignore potential changes in pore water pressure caused by ploughing particles [41,45], and begin our study at the glacier bed with an exploration of till mechanics.

### (a) Mechanical properties of till

We adopt a phenomenological model for the mechanical strength of till that depends on basal slip rate *u*_*b*_ and the state of the subglacial till *θ*. This rate-and-state friction model accounts for instantaneous basal slip rate and, importantly, basal slip history, and was derived to explain numerous laboratory measurements of sliding on bare rock and granular interfaces. Rate-and-state friction is widely used in studies of earthquake nucleation and slow-slip events on tectonic faults, and gives the instantaneous shear strength of subglacial till as [55,56]

2.1 $$\tau_{t}=N\mu =N\left[\mu_{n}+a\ln\left(\frac{u_{b}}{u_{b_{n}}}\right)+b\ln\left(\frac{\theta u_{b_{n}}}{d_{c}}\right)\right],$$

where *μ*_*n*_ is the coefficient of nominal internal friction (i.e. friction coefficient at steady state and $u_{b}=u_{b_{n}}$), *d*_*c*_ is a characteristic slip displacement, $u_{b_{n}}$ is a constant reference velocity, and the constants *a* and *b* are material parameters that define the magnitude of the direct (velocity, *u*_*b*_) and evolution (state, *θ*) effects, respectively. As we will discuss, *b* is important for this study because it encodes the effect of dilation on the bulk friction coefficient *μ*. In our idealized glacier geometry, the bed is horizontal and effective normal stress is equal to effective pressure *N*, defined as

2.2 $$N=p_{i}-p_{w},$$

with *p*_*w*_ the pore water pressure in the till and the ice overburden pressure *p*_*i*_ defined as

2.3 $$p_{i}=\rho_{i}gh,$$

where *ρ*_*i*_ is the mass density of ice and *g* is gravitational acceleration.

Rate-and-state friction has received attention in studies of the ice–bed interface [38,41,44,75,76] and is widely studied for slip on tectonic faults containing gouge [52,77–79], a material mechanistically similar to till [80]. Though distinct in many respects, earthquakes and glacier surges are analogous in the sense that both involve long quiescent periods and relatively short activation time scales. Slow slip on tectonic faults is particularly relevant to studying glacier surges because of their comparable slip durations and slow slip rates compared with major earthquakes [52,53]. Incipient motion in both earthquakes and glacier surges is brought on by excess applied stress relative to frictional resistance. While stresses and displacement rates are orders of magnitude higher in earthquakes than in glaciers, the experimentally verified rate-and-state friction model is applicable to glacier surges as there is no known lower bound on velocity for the model to be valid [81]. Furthermore, because the rate-and-state friction model given in equation (2.1) represents friction along grain boundaries within the deforming till layer, this model is consistent with the skin-friction regime in regularized Coulomb friction rules [82,83].

When till is deformed, individual grains are mobilized by cataclastic flow (which includes grain rolling and grain boundary sliding), dilation and comminution. Under small displacements, the granular structure of the till is related to the pre-deformed structure, meaning that the till essentially remembers its prior state. Memory is represented by the state variable *θ* (units of time). State has been taken to represent the product of the contact area and intrinsic strength (quality) of the contact [84], but also has been interpreted as the average age of contacts between load-bearing asperities [85]. Under either interpretation, state is expected to evolve as a function of time, slip and effective normal stress [55,85–87]. To represent the evolution of *θ*, we adopt the state evolution equation, sometimes referred to as the slip law [56]

2.4 $$\dot{\theta }=-\frac{\theta u_{b}}{d_{c}}\ln\left(\frac{\theta u_{b}}{d_{c}}\right),$$

which dictates that state evolves only in the presence of slip. The only stable steady state in equation (2.4) exists at *θ* = *d*_*c*_/*u*_*b*_; when *u*_*b*_ > 0, *θ* always tends towards the stable steady state. Increasing *u*_*b*_ beyond *d*_*c*_/*θ*—through enhanced surface meltwater flux, calving or other external forcing—will reduce *θ* over time. Similarly, when *u*_*b*_ < *d*_*c*_/*θ*, *θ* will increase towards steady state. In the next section, we show that changes in *θ* are brought about through till compaction and dilation. As such, *θ* accounts for the basal slip history and plays a key role in determining bed strength and the response of bed strength to shear and external forcing.

Steady-state till shear strength occurs when state evolution ceases ($\dot{\theta }=0$) and is defined as

2.5 $${\hat{\tau }}_{t}=N\left[\mu_{n}+(a-b)\ln\left(\frac{{\hat{u}}_{b}}{u_{b_{n}}}\right)\right],$$

where ${\hat{u}}_{b}=d_{c}/\hat{\theta }$ is the steady-state basal slip rate. (Hereafter, hatted values indicate steady state for the respective variable.) Equation (2.5) indicates that the condition for a rate-weakening friction coefficient is *b* > *a*, indicating that $\hat{\mu }({\hat{u}}_{b_{1}})<\hat{\mu }({\hat{u}}_{b_{2}})$ for ${\hat{u}}_{b_{1}}>{\hat{u}}_{b_{2}}$. The other important factor to consider in this study is *d*_*c*_, the slip distance over which state (and porosity) evolve. Computational and microphysical studies have concluded that *d*_*c*_ is proportional to the thickness of the deforming layer [79,88,89], which can be expected to be of order 0.1–1 m in subglacial till and varies with permeability [59,90]. Other factors influencing *d*_*c*_ include grain size and porosity [79].

### (b) Pore water pressure

Till shear strength is proportional to effective pressure (equation (2.1)), the difference between overburden and pore water pressure (equation (2.2)). Assuming that the mass density of ice remains constant, effective pressure can only vary during surges because of changes in ice thickness and pore water pressure. Pore water pressure is linked to till compaction and dilation through changes in the effective till porosity. Thus, if we assume that the till is always saturated, then the rate of change of water mass per unit volume within the till is given as

2.6 $${\dot{m}}_{w}=\rho_{w}\dot{\phi },$$

where *ϕ* is the (dimensionless) effective till porosity, defined as the ratio of pore volume to total volume, and *ρ*_*w*_ is the density of water. In this section, we seek to understand the rate of change in pore water pressure as a function of basal slip rate under the basic assumptions that water is incompressible over the range of reasonable subglacial pressures and that frictional heating at the ice–bed interface and plastic dissipation within the till are negligible.

#### (i) (Evolution of porosity)

Assuming that individual grains in the till are rigid, strain within the till will be accommodated by changes in porosity. Adopting an elastic–plastic model for the deformation of granular till, wherein the total strain is equal to the sum of the elastic and plastic strains, we separate porosity changes into an elastic component ${\dot{p}}_{w}\beta$ and a plastic component ${\dot{\phi }}_{p}$ such that [78,91]

2.7 $$\dot{\phi }={\dot{p}}_{w}\beta +{\dot{\phi }}_{p},$$

where

2.8*a,b* $$\beta =\frac{\partial \phi }{\partial p_{w}}=\frac{\epsilon_{e}{\left(1-\phi \right)}^{2}}{N}$$

is the till compressibility and *ϵ*_*e*_ is the elastic compressibility coefficient, taken to be in the range *ϵ*_*e*_ ∼ 10^−3^–10^−1^ [92]. Following work by Segall & Rice [78] and Segall *et al.* [53] on slow-slip events on tectonic faults, we take the plastic component of porosity to have the same form as the evolution component of the rate-and-state model for till shear strength (equation (2.1)), namely

2.9 $$\phi_{p}=\phi_{c}-\epsilon_{p}\ln\left(\frac{\theta u_{b_{n}}}{d_{c}}\right),$$

where *ϕ*_*c*_ is a (constant) characteristic porosity and *ϵ*_*p*_ is a dilatancy coefficient, a dimensionless parameter hereafter assumed constant and in the range 10^−4^ ≤ *ϵ*_*p*_ ≤ 10^−2^ [53]. We note that the only sensitivity in our model to the absolute value of *ϵ*_*p*_ is to the evolution of porosity; surge behaviour, the main focus of this study, is influenced only by the ratio *ϵ*_*p*_/*β*, which represents the relative importance of each term in equation (2.7). By adopting equation (2.9), we are assuming that plastic deformation of the till is completely determined by changes in state, *θ*, the only variable in equation (2.9). This assumption is physically justifiable: irreversible changes in porosity necessitate a change in the average age of granular contacts and, equivalently, a change in the product of the contact area and quality, both of which are the physical interpretations of state (*θ*) discussed above. Differentiating equation (2.9) in time yields

2.10 $${\dot{\phi }}_{p}=-\epsilon_{p}\frac{\dot{\theta }}{\theta },$$

an expression that indicates that shearing of the till layer causes it to compact (${\dot{\phi }}_{p}<0$) when *θ* is below steady state (*θ* < *d*_*c*_/*u*_*b*_) and to dilate when *θ* is above steady state. Such behaviour is consistent with observations of the response of over- and under-consolidated soils to shear [50]. This relationship between plastic till deformation and state gives rise to rich mechanical relationships between compaction, dilation and shearing, as is expected from sediments.

#### (ii) (Evolution of pore water pressure)

Let us now consider water flux in the till in response to changes in porosity and sources outside the till shear layer. The rate of change of water mass is given by plugging the expressions for the total rate of change in porosity (equations (2.4), (2.7) and (2.10)) and the rate of irreversible (plastic) change in porosity (equation (2.10)) into the expression for the rate of change in mass per unit volume (equation (2.6)), yielding

2.11 $${\dot{m}}_{w}=\rho_{w}{\dot{p}}_{w}\beta +\rho_{w}\epsilon_{p}\frac{u_{b}}{d_{c}}\ln\left(\frac{\theta u_{b}}{d_{c}}\right).$$

Conservation of water mass gives

2.12 $$\frac{\partial q_{w}}{\partial z}+{\dot{m}}_{w}=0,$$

where *q*_*w*_ is the vertical water mass flux. Here, we have assumed that horizontal gradients in water pressure are negligible compared with vertical gradients and the bed slope is sufficiently shallow to allow us to consider only vertical water flux. Taking the basal ice to be impermeable then requires water flux to be entirely into and out of the deforming till layer. Under these conditions, Darcy’s law is given as

2.13 $$q_{w}=-\frac{\rho_{w}\gamma_{h}}{\eta_{w}}\frac{\partial p_{w}}{\partial z},$$

where *γ*_*h*_ is the till permeability and *η*_*w*_ is the dynamic viscosity of water. Combining equations (2.11)–(2.13) under the assumption that till permeability is spatially constant and independent of porosity gives

2.14 $${\dot{p}}_{w}=\kappa_{h}\frac{\partial^{2}p_{w}}{\partial z^{2}}+\frac{\epsilon_{p}\;\dot{\theta }}{\epsilon_{e}\;\theta }\frac{N}{{\left(1-\phi \right)}^{2}},$$

where

2.15 $$\kappa_{h}=\frac{\gamma_{h}}{\eta_{w}\beta }$$

is the hydraulic diffusivity of the deforming till layer. Measurements of hydraulic diffusivity in till give a range for *κ*_*h*_ of approximately $10^{-9}\text{--}10^{-4}\;{\text{m}}^{2}\;{\text{s}}^{-1}$, with a strong sensitivity to clay content [93,94]. We take constant effective permeability to be a reasonable first approximation given the small change in permeability under glaciologically relevant pressures and strains found in discrete-element modelling studies [90]. A more general treatment of pore water pressure evolution would include a porosity-dependent permeability in place of a constant effective permeability—for example, the Kozeny–Carman model used by Clarke [39]. We reserve this additional complexity for future work as our simple model retains the salient physical processes.

Shearing in till concentrates in a thin, multi-layer zone that is typically several centimetres thick [60,95–97]. We, therefore, approximate

2.16 $$\frac{\partial^{2}p_{w}}{\partial z^{2}}=\frac{p_{w_{\infty }}-2p_{w}+p_{w_{r}}}{h_{s}^{2}},$$

where *h*_*s*_ is the thickness of the shear zone in the till, $p_{w_{\infty }}$ is the water pressure in the underlying permeable half-space and $p_{w_{r}}$ is the water pressure in the basal hydrological system (figure 1). With this approximation, equation (2.14) becomes

2.17 $${\dot{p}}_{w}=\frac{p_{w_{\infty }}-2p_{w}+p_{w_{r}}}{t_{h}}+\frac{\epsilon_{p}\;\dot{\theta }}{\epsilon_{e}\;\theta }\frac{N}{{\left(1-\phi \right)}^{2}},$$

where the first term represents Darcian flow into and out of the deforming till layer and the second term represents dynamical (dilation-driven) changes in pore water pressure. The Darcy flow component of pore water pressure evolution is inversely proportional to the characteristic diffusive time scale for pore water in the deforming till layer

2.18 $$t_{h}=\frac{h_{s}^{2}}{\kappa_{h}}.$$

To simplify the analysis, we hereafter take *t*_*h*_ to be constant, thereby ignoring the dependence of *κ*_*h*_ and *h*_*s*_ on effective pressure *N* and porosity *ϕ*. We justify this simplification by noting that *κ*_*h*_ (equation (2.15)) and till thickness *h*_*s*_ roughly scale as *N*, though a detailed analysis of the relation between *h*_*s*_ and *N* is beyond the scope of this work [39]. Assuming *h*_*s*_ ∼ *N* and *κ*_*h*_ ∼ *N*, we can suppose, to a reasonable approximation, *t*_*h*_ ∼ *N*, which should retain the same order of magnitude during incipient surge motion. Similarly for permeability, where compaction-driven reductions in permeability will induce relatively small (factor of 2) decreases in thickness *h*_*s*_ [90]. Such small changes are unlikely to dramatically alter the dynamics of surge motion captured here, and we leave for future work a more detailed analysis involving variable *t*_*h*_.

From the second term in equation (2.17), we can see that the sign of the dynamical (or dilation-driven) component of ${\dot{p}}_{w}$ is determined by the state of the till. When state *θ* is below (above) steady state and *t*_*h*_ > 0, pore water pressure will increase (decrease) until steady state is achieved. These changes in pore water pressure are entirely due to changes in till porosity: compaction (${\dot{\phi }}_{p}<0$) results in non-zero rates of change in the dynamical component of water pressure because the second term in equation (2.17) is $\epsilon_{p}\dot{\theta }N/[\epsilon_{e}\theta {\left(1-\phi \right)}^{2}]=-{\dot{\phi }}_{p}/\beta$. Whether *p*_*w*_ decreases or increases following step changes in basal slip rate depends on whether the ratio *θu*_*b*_/*d*_*c*_ is greater than or less than unity. Equation (2.17) also shows that steady-state pore water pressure is ${\hat{p}}_{w}=p_{w_{\infty }}=p_{w_{r}}$ when $\dot{\theta }=0$.

### (c) Basal slip acceleration

Glacier ice is an incompressible viscous fluid in laminar flow, and the momentum equation, incompressibility condition and continuity equation, respectively, take the forms

2.19 $$\begin{array}{rl} & 0=\frac{\partial \tau_{ij}}{\partial x_{j}}-\frac{\partial \tilde{p}}{\partial x_{i}}+\rho_{i}g\delta_{iz}, \end{array}$$

2.20 $$\begin{array}{rl} & 0=\frac{\partial u_{i}}{\partial x_{i}} \end{array}$$

2.21 $$\begin{array}{rl} \;\text{and}\; & \dot{h}=\dot{M}-\frac{\partial }{\partial x_{i}}(h{\bar{u}}_{i}), \end{array}$$

where *u*_*i*_ is the ice velocity vector, ${\bar{u}}_{i}$ is the depth-averaged ice velocity vector, *τ*_*ij*_ is the deviatoric stress tensor, *δ*_*ij*_ is the Kronecker delta, $\tilde{p}$ is the mean isotropic ice stress (pressure), $\dot{M}$ is the total surface mass balance (which includes surface and basal mass balance and is positive for mass accumulation), and we employ the summation convention for repeated indices. To simplify our analysis, we neglect vertical shearing in the ice column and adopt a depth-integrated momentum equation (often referred to as the shallow-shelf approximation) [98]

2.22 $$2\frac{\partial }{\partial x}(h\tau_{xx})+\frac{\partial }{\partial y}(h\tau_{xy})+\tau_{b}=\tau_{d},$$

where *τ*_*xx*_ is the extensional deviatoric stress, *τ*_*xy*_ is the lateral shear stress and we have neglected the transverse normal (deviatoric) stress *τ*_*yy*_. In some surge-type glaciers, vertical shearing may be the dominant flow regime during the quiescent phase, while basal slip is the dominant flow regime during the surge phase. Equation (2.22) is valid only when basal slip is dominant, and thus a model of basal slip acceleration derived from equation (2.22) may not fully detail glacier flow during incipient surge acceleration in some glaciers. Nevertheless, this simplification is reasonable because the focus of this work is on till mechanics and the flow model based on equation (2.22) will represent the salient processes of nascent surge acceleration. We reserve for future work a more detailed analysis that retains more components of the stress divergence and is able to capture the transition from vertical-shear-dominated flow to basal-slip-dominated flow.

Force balance dictates that basal shear traction cannot exceed the lesser of applied stress and yield stress of the till, giving rise to the relation [19,46]

2.23 $$\tau_{b}=\min(\tau_{d},\tau_{t}),$$

where *τ*_*t*_ = *μN* is the till shear strength (equation 2.1) and the gravitational driving stress is defined as

2.24 $$\tau_{d}=\rho_{i}gh\alpha ,$$

where *α* is the ice surface slope, assumed small such that sin(*α*) ≈ *α*. Recall that we are focusing on the case in which rapid flow during the surge is accommodated primarily by deformation of the bed, giving rise to the relations *τ*_*b*_ = *τ*_*t*_ and *u*_*s*_ ≈ *u*_*b*_, where *u*_*s*_ is the ice-surface velocity. We note that equation (2.23) is consistent with the so-called regularized Coulomb sliding law, which has recently emerged as a candidate for a universal form of the sliding law, because, in this work, we are focused on the skin-friction regime defined by equation (2.1) [74,82,83,99].

Let us now focus only on the region where the surge is initiated and assume the areal extent of incipient surge motion is large enough to make the gradient of longitudinal stress (first term in equation (2.22)) negligible during the nascent surge phase. Taking ice to be a shear-thinning (i.e. pseudoplastic) viscous fluid, the constitutive relation, commonly known as Glen’s law [100], is

2.25 $${\dot{\varepsilon }}_{e}=A\tau_{e}^{n},$$

where ${\dot{\varepsilon }}_{e}=\sqrt{{\dot{\varepsilon }}_{ij}{\dot{\varepsilon }}_{ij}/2}$ is the effective strain rate, $\tau_{e}=\sqrt{\tau_{ij}\tau_{ij}/2}$ is the effective deviatoric stress, the rate factor *A* is a scalar and the stress exponent is *n* = 3. Hereafter, *A* and *n* are assumed constant. Under our prior assumptions, $2{\dot{\varepsilon }}_{e}\approx \partial u_{b}/\partial y$ and *τ*_*e*_ ≈ *τ*_*xy*_. Integrating the reduced form of equation (2.22) twice along *y* subject to the symmetry condition *τ*_*xy*_ = 0 at the centreline and no-slip condition at the margins gives the centreline basal slip rate [94]

2.26 $$u_{b}=\frac{2A{\left(\rho_{i}g\right)}^{n}w^{n+1}}{n+1}{\left[\alpha -\mu \left(1-\frac{p_{w}}{p_{i}}\right)\right]}^{n}.$$

Taking *w* to be constant and differentiating equation (2.26) with respect to time yields an expression for acceleration of basal slip

2.27 $${\dot{u}}_{b}=nu_{b}\left[\frac{\dot{\alpha }-\mu \frac{p_{w}}{p_{i}}\left(\frac{\dot{h}}{h}-\frac{{\dot{p}}_{w}}{p_{w}}\right)-b\frac{\dot{\theta }}{\theta }\left(1-\frac{p_{w}}{p_{i}}\right)}{\alpha +(an-\mu )\left(1-\frac{p_{w}}{p_{i}}\right)}\right],$$

where the rates of change in glacier geometry ($\dot{h}$ and $\dot{\alpha }$), pore water pressure (${\dot{p}}_{w}$) and state ($\dot{\theta }$) all contribute to the basal slip acceleration, along with instantaneous geometry (*h* and *α*), pore water pressure (*p*_*w*_), state (*θ*) and basal slip rate (*u*_*b*_). Note that the conditions discussed and imposed in the model development—*τ*_*d*_ > *τ*_*b*_ (glacier is slipping at the bed), *τ*_*b*_ = *τ*_*t*_ (the till is deforming) and *p*_*w*_ < *p*_*i*_ (till has non-zero shear strength)—ensure that the denominator in equation (2.27) is always greater than zero.

Equation (2.27) is the central result of this study. This formula describes the dependence of surge acceleration on glacier geometry, pore water pressure and the properties of the till. The terms in the numerator can be related to the processes of interest during the surge. Namely, the first term in the numerator ($\dot{\alpha }$) essentially represents the rate of change in the gravitational driving stress. The second term in the numerator captures the evolution of effective pressure (*N*), which governs the shear strength of the bed. The third and final term in the numerator accounts for the influence of dilation on the internal friction coefficient of the till. We spend the remainder of this study investigating the influence of the various physical processes represented in equation (2.27).

## 3. Results

Since shear strength of the till is the governing factor in surge motion and is defined by three variables (overburden pressure *p*_*i*_, pore water pressure *p*_*w*_ and the internal friction coefficient *μ*), we present the results in three sections. In the first section, we discuss the evolution of pore water pressure following an increase in basal slip rate. Second, we consider the acceleration of basal slip for a glacier with a fixed geometry (i.e. fixed overburden pressure). Last, we explore the full model, which allows for variations in pore water pressure, glacier geometry and internal friction coefficient for till.

### (a) Evolution of pore water pressure

**Figure 2.**
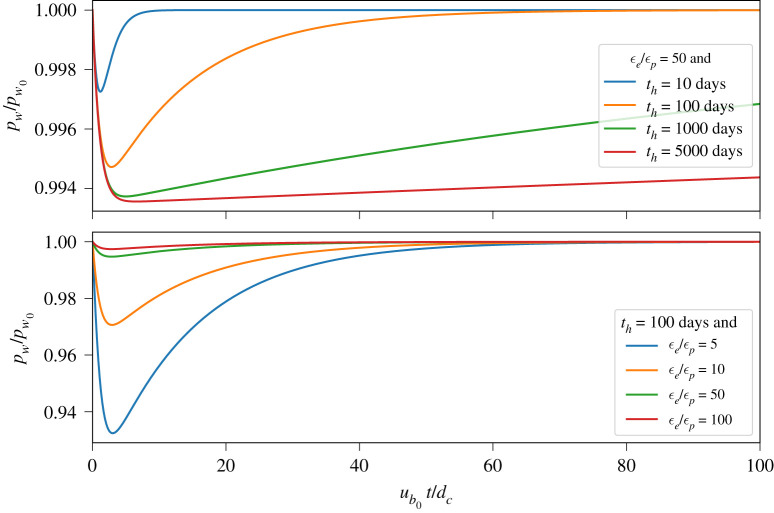
Evolution of pore water pressure in the deforming till layer (§3a) following a step increase in basal slip rate, $u_{b}=10u_{b_{0}}$ for *t* ≥ 0, from an initial steady state (${\hat{\theta }}_{0}=d_{c}/u_{b_{0}}$). (*a*) The influence of the hydraulic diffusion time scale of till on the evolution of pore water pressure for a fixed *ϵ*_*e*_/*ϵ*_*p*_ ratio while (*b*) illustrates the influence of the ratio of the elastic to the plastic compressibility coefficients for a fixed diffusion time scale. Water pressures in the subglacial hydrological system ($p_{w_{r}}$) and underlying stagnant till layer ($p_{w_{\infty }}$) are defined as $p_{w_{r}}=p_{w_{\infty }}=0.9p_{i}$ and held constant in time. Other relevant parameters values are: *d*_*c*_ = 0.1 m, *μ*_*n*_ = 0.5, $u_{b_{0}}=10\;\text{m/yr}$, ${\hat{\phi }}_{0}=0.1$ and ${\hat{p}}_{w_{0}}=p_{w_{r}}=p_{w_{\infty }}$. (Online version in colour.)

Pore water pressure in the deforming till layer evolves through dilation and compaction of the till as well as through the exchange of water between the deforming till layer, the subglacial hydrological system and the stagnant till layer that underlies the deforming layer (equation (2.17) and figure 2). In our model, the pressures in the stagnant till layer ($p_{w_{\infty }}$) and the subglacial hydrological system ($p_{w_{r}}$) are assumed constant in time, and the flow of water into or out of the deforming till layer is described by Darcy’s law (equation (2.13)). Using the parameter values given in the caption of figure 2, we integrate equations (2.4), (2.7) and (2.17) forwards in time from the (steady-state) initial conditions $u_{b_{0}}=10\;\text{m/yr}$, ${\hat{\phi }}_{0}=0.1$, ${\hat{\theta }}_{0}=d_{c}/u_{b_{0}}$ and ${\hat{p}}_{w_{0}}=p_{w_{r}}=p_{w_{\infty }}$ using the variable-coefficient ordinary differential equation (VODE) solver implemented in SciPy (v. 1.3.1), an open-source Python toolkit [101].

The results shown in figure 2 illustrate how the evolution of pore water pressure *p*_*w*_ following a step increase in basal slip rate ($u_{b}=10u_{b_{0}}$) is influenced by the hydraulic diffusion time scale of the deforming till layer (*t*_*h*_) and the relative values of the elastic (*ϵ*_*e*_) and plastic (*ϵ*_*p*_) compressibility coefficients. Note that, because we hold *t*_*h*_ fixed in time, only the relative compressibility ratio *ϵ*_*e*_/*ϵ*_*p*_ influences pore water pressure, not the absolute values of *ϵ*_*e*_ and *ϵ*_*p*_. All cases shown in figure 2 start at steady state and indicate initial decreases in pore water pressure *p*_*w*_ in response to till dilation followed by a return to steady state (${\hat{p}}_{w}={\hat{p}}_{w_{0}}=p_{w_{r}}=p_{w_{\infty }}$) via Darcian flow over a time scale proportional to the diffusion time scale (cf. equation (2.17)). The minimum pore water pressure is determined by the diffusion time scale *t*_*h*_ and the relative compressibility *ϵ*_*e*_/*ϵ*_*p*_. For a given relative compressibility, longer diffusion time scales, corresponding to lower till permeabilities, lead to a greater drop in pore water pressure (figure 2*a*). For a given diffusion time scale, smaller values of relative compressibility, which indicate stronger dilatancy of the till relative to poroelastic effects, result in greater drops in pore water pressure (figure 2*b*).

### (b) Acceleration with fixed ice thickness

We now consider glacier acceleration. As a first step, we simplify our analysis by assuming that the time scale of interest is longer than the time scale for pore water diffusion (*t* > *t*_*h*_) but short enough to allow us to reasonably neglect changes in glacier geometry. While it can be argued that this condition may be physically contrived in some cases, it is useful for exploring surge dynamics and the behaviour of the till in the absence of some complicating factors (in the next section, we will allow glacier geometry to evolve). After fixing glacier geometry by imposing $\dot{h}=0$ and $\dot{\alpha }=0$ at all times, we solve the system of equations defined by equations (2.4), (2.7), (2.17) and (2.27). For all results discussed here, we prescribe as the initial velocity $u_{b}=1.1u_{b_{0}}$ at *t* = 0, where ${\hat{u}}_{b_{0}}=10\;\text{m/yr}$, and set the initial values for all other variables to their respective steady-state values. The system of equations is stiff; therefore, we integrate forwards in time using an implicit Runge–Kutta method—specifically, the Radau IIA fifth-order method—implemented in SciPy (v. 1.3.1).

**Figure 3.**
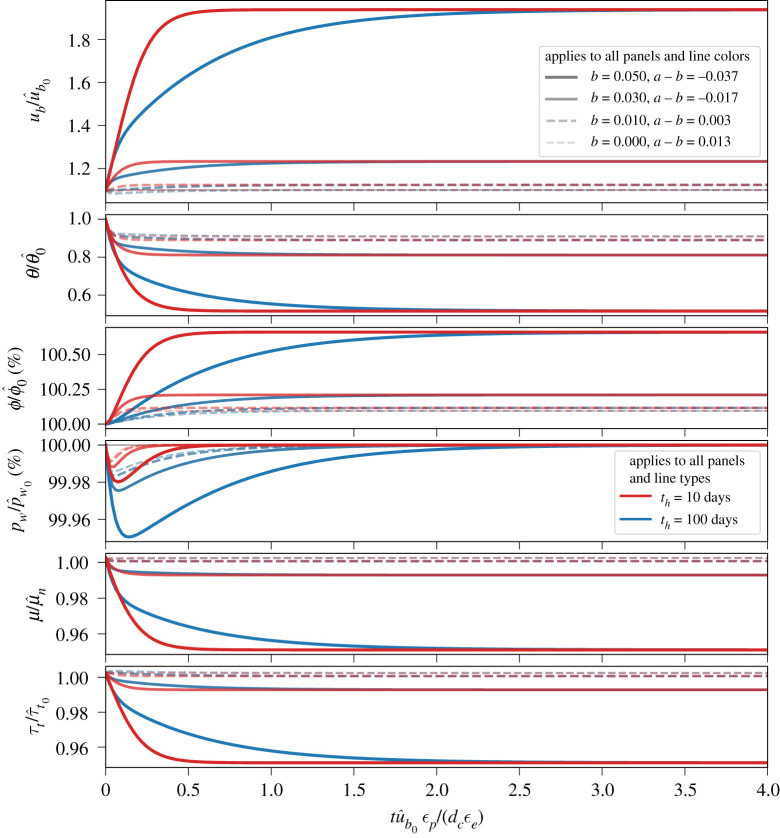
Evolution of (from top to bottom) basal slip rate (*u*_*b*_), state (*θ*), porosity (*ϕ*), pore water pressure in the deforming till layer (*p*_*w*_), internal friction coefficient for till (*μ*) and till shear strength (*τ*_*t*_) following a perturbation in basal slip rate from steady state for fixed ice thickness and surface slope (§3b). The perturbation in basal slip is $u_{b}=\lambda {\hat{u}}_{b_{0}}$ at *t* = 0 with *λ* = 1.1, a value indicated by the thin solid grey line in the upper panel. We consider a range of evolution effects (*b* values, indicated by line widths and intensities in all panels) and two hydraulic diffusion time scales: $t_{h}=10\;\text{days}$ (red lines in all panels) and $t_{h}=100\;\text{days}$ (blue lines in all panels). In all panels, solid lines indicate rate weakening (*a* < *b*) and dashed lines indicate rate strengthening (*a* > *b*). Prescribed values are ${\hat{u}}_{b_{0}}=10\;\text{m/yr}$, ${\hat{p}}_{w}/p_{i}=0.92$, ${\hat{\phi }}_{0}=0.1$, *d*_*c*_ = 0.1 m, *ϵ*_*p*_ = 10^−3^, *ϵ*_*e*_ = 50*ϵ*_*p*_, *n* = 3, *α* = 0.05, *a* = 0.013 and *μ*_*n*_ = 0.5. (Online version in colour.)

In the cases shown in figure 3, we focus on the influences of a range of viable evolution effects (*b* values; indicated by line intensity and thickness) and different hydraulic diffusion time scales (*t*_*h*_; indicated by colours). Aside from *b* and *t*_*h*_, all parameters are the same for all cases and are listed in the caption to figure 3. Note that *a* = 0.013, so in terms of the till friction coefficient *μ*, the cases shown in figure 3 are both rate weakening (*a* < *b*; solid lines) and rate strengthening (*a* > *b*; dashed lines).

The most notable feature in all cases shown in figure 3 is the lack of unstable acceleration. Steady-state speed is governed by the steady-state shear strength of till (equation (2.5)) and is therefore sensitive to the rate-and-state parameters (*a* − *b*) and *μ*_*n*_. Since the direct effect (*a*) is constant in all cases in figure 3, increasing the evolution effect (*b*) leads to a greater steady-state stress drop and faster steady-state basal slip rate because of the increasingly negative value (*a* − *b*). The steady-state values for all state variables are independent of the diffusion time scale *t*_*h*_ and characteristic slip length *d*_*c*_. The primary influences of *t*_*h*_ and *d*_*c*_ are on the time the system takes to reach steady state and the peak change in pore water pressure. These results show that the system tends to steady state over a characteristic time scale that scales with the (dimensionless) hydraulic transmittance

**Figure 4.**
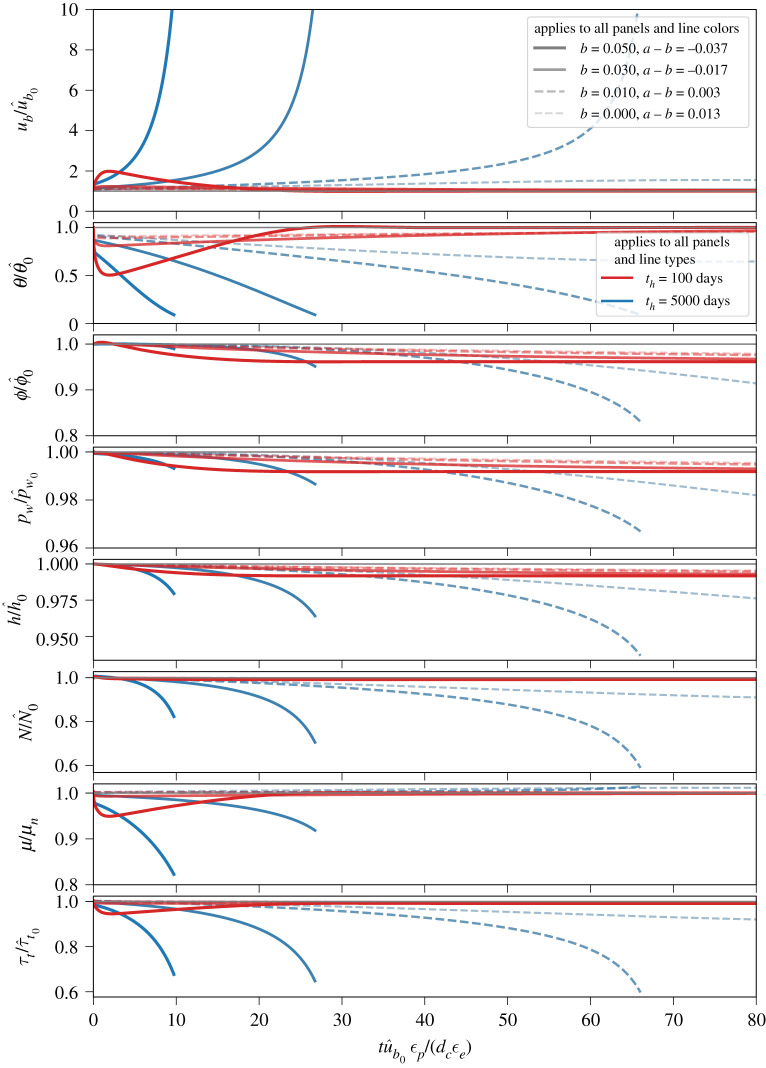
Evolution of (from top to bottom) basal slip rate (*u*_*b*_), state (*θ*), porosity (*ϕ*), pore water pressure in the deforming till layer (*p*_*w*_), ice thickness (*h*), effective pressure (*N*), internal friction coefficient for till (*μ*) and till shear strength (*τ*_*t*_) following a perturbation in basal slip rate from steady state for variable ice thickness (§3c). All factors are normalized by their respective initial steady-state values. Velocity perturbation and other parameters are the same as for figure 3. Line thickness and continuity indicate different values of the evolution term *b*, as indicated in the legend in the upper panel, while line colours indicate values of the hydraulic diffusivity time scale for till (*t*_*h*_), as shown in the legend in the second panel. Dashed lines indicate that the internal friction coefficient is rate strengthening (i.e. (*a* − *b*) > 0). Truncated lines occur when the integration is stopped; we chose $u_{b}/{\hat{u}}_{b_{0}}=10$, which we define as indicating a surge, as the stopping condition. Over a long enough time scale, the line representing *b* = 0 and *t*_*h*_ = 5000 days eventually surges. (Online version in colour.)

**Figure 5.**
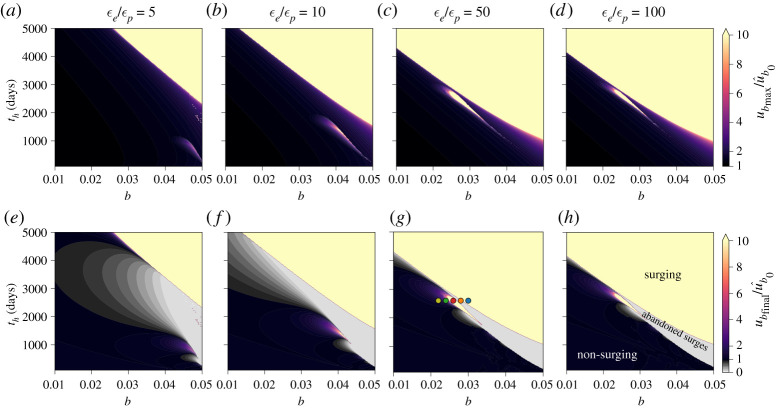
Parameter space covering the three principal parameters influencing incipient surge motion: the evolution effect *b* (*x*-axes of all panels), hydraulic diffusion time scale *t*_*h*_ (*y*-axes of all panels) and relative till compressibility *ϵ*_*e*_/*ϵ*_*p*_ (columns). The top row (*a*–*d*) indicates the maximum basal slip rate ($u_{b_{\text{max}}}/{\hat{u}}_{b_{0}}$) achieved by the modelled glacier following a perturbation identical to that in figure 4, while the bottom row (*e*–*h*) shows the final basal slip rate ($u_{b_{\text{final}}}/{\hat{u}}_{b_{0}}$). Coloured dots in (*g*) show the line colours and parameters for model outputs shown in figure 6. All other parameters are the same as in figure 4. (Online version in colour.)

**Figure 6.**
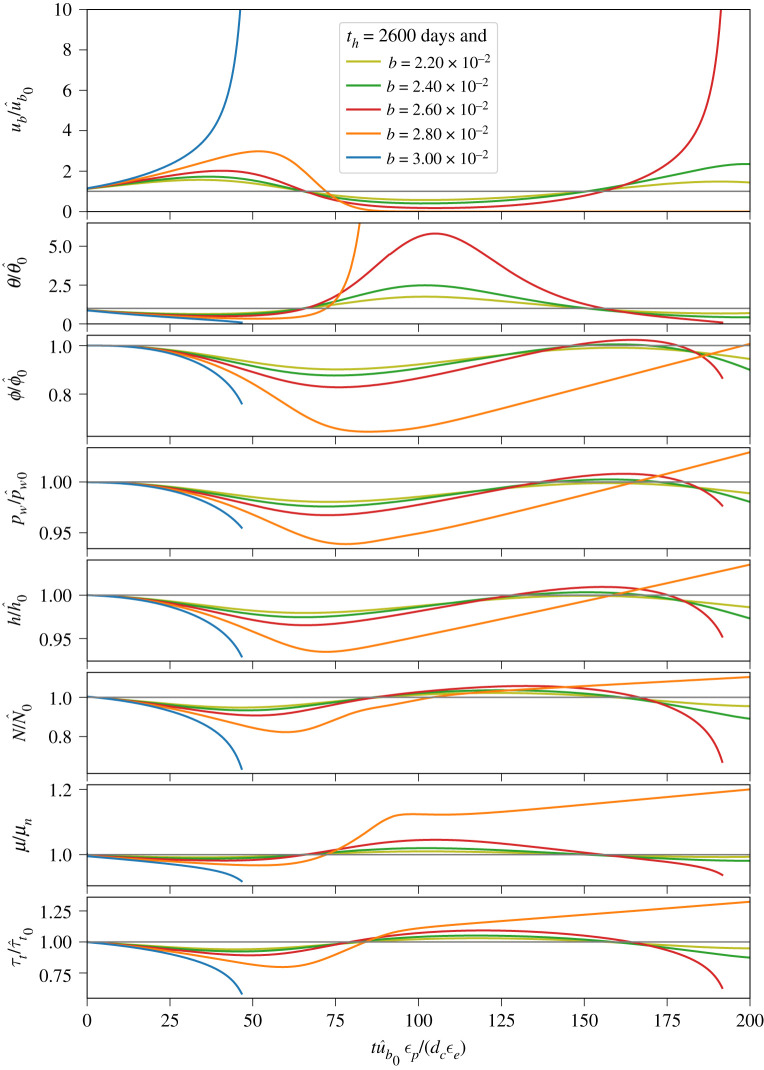
Similar to figure 4 except that models are run using parameter values indicated in figure 5*g*. Line colours correspond to dot colours in figure 5*g*. (Online version in colour.)

**Figure 7.**
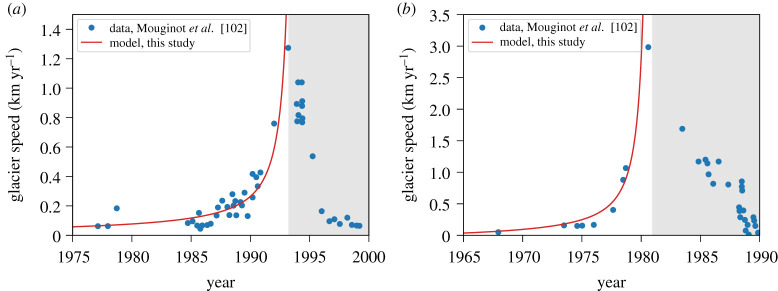
Comparison between our model and observed glacier surface velocities from two surges, (*a*) *L. Bistrup Bræ* and (*b*) Storstømmen, northeast Greenland [102]. Model parameters are the same as in figures 3 and 4, and with *b* = 0.03, *t*_*h*_ = 3000 days and initial velocity set according to the data. The greyed regions indicate the slowdown phase of the surge, which our model does not attempt to represent. (Online version in colour.)

3.1 $$\psi_{0}=\frac{\epsilon_{p}\lambda {\hat{u}}_{b_{0}}t_{h}}{\epsilon_{e}d_{c}},$$

where *λ* is the (dimensionless) perturbation in *u*_*b*_ (*λ* = 1.1 in figures 3–7). Equation (3.1) is defined as the ratio of the hydraulic diffusion time scale *t*_*h*_ to the time scale for dilation-driven changes in pore water pressure $d_{c}\epsilon_{e}/(\epsilon_{p}{\hat{u}}_{b_{0}})$, which follow from the coefficients in equation (2.17). The dependence on *ψ*_0_ of the time to steady state is indicated in figure 3 by noting that the only term in *ψ*_0_ that changes between the different cases is the *t*_*h*_. The time axes in figure 3 are normalized by $d_{c}\epsilon_{e}/(\epsilon_{p}{\hat{u}}_{b_{0}})$, the time scale for dilation-driven changes in pore water pressure, to help show that model realizations in which the diffusion time scale *t*_*h*_ is an order of magnitude longer take an order of magnitude longer time to evolve to steady state. As we show in the next section, the time required to reach steady state is a crucial factor governing whether or not a glacier surges.

The behaviour of the model in the absence of changes in glacier geometry (figure 3) provides further insight that helps to explain some of the results of the full model presented in the next section. For instance, the till dilates in all cases because of initial step and subsequent changes in basal slip rate (figure 3). The amplitude of the change in till porosity scales with the evolution parameter *b*, with larger values of *b* resulting in greater dilatancy. As seen in the previous section, higher dilatancy results in a larger drop in pore water pressure as the glacier accelerates. Dilatancy also drives a reduction in the internal friction coefficient of till, as a dilated till provides less resistance to shearing owing to reduced contact areas between grains. This drop in the internal friction coefficient commensurately reduces the shear strength of the till.

### (c) Acceleration with variable ice thickness

Over longer time scales, dynamically driven changes in the glacier geometry can be important, and we must consider the full expression given in equation (2.27). To do so, we approximate changes in glacier geometry by recalling that *h* varies only in the along-flow (*x*) direction and focusing only on the central trunk of the glacier where across-flow variations in velocity can be neglected. Thus, the continuity equation (equation (2.21)) becomes

3.2 $$\dot{h}=\dot{M}-\frac{\partial }{\partial x}(\zeta hu_{s}),$$

where $\zeta =\bar{u}/u_{s}$, $\bar{u}$ is the depth-averaged glacier speed and *u*_*s*_ is the glacier surface speed. Since we have taken ice to be a non-Newtonian viscous fluid, we have (*n* + 1)/(*n* + 2) ≤ *ζ* ≤ 1, where *n* is the stress exponent in the constitutive relation for ice (equation (2.25)) [103]. In this study, we adopt the most common value for the stress exponent, *n* = 3, and we prescribe *ζ* = 1 for consistency with the reduced momentum equation in equation (2.22) (when *ζ* = 1, *u*_*s*_ = *u*_*b*_). We further simplify the expression for dynamical thinning by neglecting extensional strain rates (consistent with the assumptions in §2c), yielding

3.3 $$\dot{h}\approx \alpha \zeta (u_{*}-u_{s}),$$

where $u_{*}=\dot{M}/(\alpha \zeta )$ is the balance velocity. Finally, the rate of change in surface slope becomes

3.4*a,b* $$\dot{\alpha }\approx -\frac{\partial \dot{h}}{\partial x}\approx \frac{\dot{h}\alpha }{h},$$

where equation (3.4*b*) follows from the assumption of a parabolic surface profile for the glacier [92]. These approximations complete the quasi-one-dimensional model, and we solve equations (2.4), (2.17), (2.27), (3.3) and (3.4) using the same parameters, initial conditions and numerical solver as in §3b.

The results, shown in figure 4, indicate markedly different behaviour from the case where glacier geometry was held fixed (§3b). Most notably, surging—defined here as an order of magnitude increase in basal slip rate—occurs for some combinations of the evolution parameter *b* and diffusion time scale *t*_*h*_. In particular, for our chosen parameters (given in the caption to figure 4), higher *b* values and longer *t*_*h*_ times result in surges. On the other hand, *b* values and *t*_*h*_ times too small and/or short to generate surge behaviours produce prosaic glacier dynamics (small *b*, short *t*_*h*_) or abandoned surges (small *b*, long *t*_*h*_), the latter of which we define as a period of rapid flow speeds (factor of 2 or more faster than quiescent speeds) that do not meet the definition of a surge, followed by a slowdown and evolution to steady state. To clarify the distinction: initial acceleration is unstable in surges and stable in abandoned surges.

To explore the processes that govern whether a surge develops, is abandoned or is essentially absent, let us focus on some illustrative cases shown in figure 4. We start with two prominent cases: those with the highest *b* values (and therefore the heaviest lines in figure 4) and different hydraulic diffusivities (i.e. *t*_*h*_ values). The case with *b* = 0.05 and higher diffusivity (and, consequently, higher hydraulic permeability and shorter *t*_*h*_), shown with the thick red lines in figure 4, undergoes an abandoned surge, defined by a brief acceleration phase, resulting in a maximum velocity of approximately twice the steady-state slip rate ($u_{b}/{\hat{u}}_{b_{0}}\approx 2$), followed by deceleration and evolution to steady state. In this case, the glacier thins somewhat, but the till tends to steady state before there is any marked change in the effective pressure at the bed (*N*). The case with *b* = 0.05 and lower hydraulic diffusivity (thick blue line) surges, with muted acceleration (relative to the case with higher hydraulic diffusivity) preceding a continual reduction in state, pore water pressure, ice thickness and till internal friction coefficient. The rates of change in each of these values when the integration was terminated (at $u_{b}/{\hat{u}}_{b_{0}}=10$) show that the glacier would continue to accelerate in the absence of contravening processes, such as increases in extensional stresses, that are not considered in our model but could manifest in a natural glacier. It is important to note that the effective pressure *N* continually decreases despite reductions in pore water pressure *p*_*w*_ because of the dynamic thinning of the glacier. In other words, reductions in overburden pressure *p*_*i*_ = *ρ*_*i*_ *g h* outpace reductions in pore water pressure *p*_*w*_, leading to a net decrease in *N* = *p*_*i*_ − *p*_*w*_ that complements reductions in the friction coefficient *μ*, ensuring that basal drag (*τ*_*b*_ = *τ*_*t*_ = *Nμ*) diminishes in time. Sustained acceleration of the glacier unequivocally indicates that the decline in basal drag outpaces thinning-induced reductions in gravitational driving stress.

Other cases shown in figure 4 indicate the same basic behaviour: till with higher values of hydraulic permeability (shorter *t*_*h*_) allows for faster acceleration, which causes the till to evolve to steady state before significant thinning of the glacier can occur. Rates of acceleration and evolution to steady state are slower in less permeable till, allowing rapid ice flow to persist for longer periods of time, facilitating dynamic thinning of the glacier. Longer time scales with relatively muted acceleration allow for thinning because dynamic glacier thinning scales as the time integral of ice velocity (equation (3.3)), meaning that longer periods of moderately rapid flow can produce more thinning than much shorter periods of somewhat faster flow. These results suggest that it is the reduction in overburden pressure *p*_*i*_, and therefore effective pressure *N*, through dynamic thinning that is ultimately responsible for sustaining surge motion. The lack of unstable acceleration when glacier geometry is fixed in time (discussed in the previous section) and the manifestation of surging in cases of rate-strengthening friction coefficients (dashed lines in figure 4) both serve to highlight the importance of dynamic thinning for sustaining surge motion.

The evolution of till porosity when the glacier geometry is allowed to vary (figure 4) is markedly different from the case with fixed glacier geometry (previous section). With a fixed glacier geometry (constant overburden pressure), till consistently dilated because the effective pressure initially decreased and then returned to steady state along with water pressure. But when we allow the glacier to thin (thereby decreasing overburden), the dependence of the rate of change in porosity on the effective pressure (via *β*; equations (2.7) and (2.8)) results in compression of the till. As the effective pressure decreases as a result of thinning of the glacier, the rate of change in porosity becomes increasingly sensitive to changes in pore water pressure (cf. equation (2.8)). Since pore water pressure decreases in response to the evolution of till state (equation (2.17)), till compaction lags reductions in pore water pressure.

The results discussed in this section indicate that the principal factors governing the surge behaviour of a glacier are the hydraulic diffusion time scale of the deforming till layer *t*_*h*_, the relative compressibility *ϵ*_*e*_/*ϵ*_*p*_, and the evolution parameter *b*, the last of which dictates the response of the internal friction coefficient to till dilation. We explore this parameter space in figure 5; except where indicated, model parameters are the same as for figure 4, and we use the same numerical solver. The results in figure 5 show that, for any relative compressibility *ϵ*_*e*_/*ϵ*_*p*_, surge-type behaviour is favoured in glaciers with high *b* values and long diffusion time scales (i.e. relatively impermeable beds). Higher *b* values imply a greater reduction in the internal friction coefficient of till (*μ*) in response to changes in porosity (and, therefore, state), with rate-weakening values (*b* > *a*) resulting in a reduced steady-state friction coefficient. Positive glacier acceleration is generally expected as the friction coefficient decreases in response to state evolution, causing surges to be favoured at higher *b* values. As previously discussed, longer diffusion time scales (i.e. lower hydraulic permeability) diminish the rate of porosity (state) evolution, and, therefore, slow the increase in effective pressure caused by dilation-driven reductions in pore water pressure (i.e. dilatant hardening). Thus, slow diffusion of pore water enables a longer acceleration period that allows time for dynamic glacier thinning to drive a net reduction in the effective pressure. Surge-type glaciers are more likely to manifest in tills that have a high relative compressibility, *ϵ*_*e*_/*ϵ*_*p*_ > 10, as these higher values imply less dilatant hardening (the reduction in pore water pressure due to shearing; cf. figure 2).

The rich dynamical behaviour illuminated in figure 5 is enhanced by the manifestation of regions (in the parameter space) of abandoned surges adjacent to the regions of surging behaviour. Abandoned surge regions are indicated in figure 5 by maximum basal slip rates greater than the initial value ($u_{b_{max}}/{\hat{u}}_{b_{0}}>2$, as shown in purple-to-red hues) and final basal slip rates less than the initial value ($u_{b_{\text{final}}}/{\hat{u}}_{b_{0}}<1$, as shown in grey tones). Abandoned surges manifest only where *b* values are relatively large but not large enough to produce a surge and diffusion time scales are slightly too short to allow for a full surge. According to our results, it is possible for a glacier to exhibit abandoned surges for any value of *ϵ*_*e*_/*ϵ*_*p*_, but the region in the parameter space that produces abandoned surges shrinks with increasing *ϵ*_*e*_/*ϵ*_*p*_ (i.e. as dilatant hardening decreases).

Two other remarkable and persistent features of the parameter space are worth highlighting. First, abandoned surge regions are accompanied by a region in the parameter space that takes the shape of an aerofoil and contains points suitable for surge-type glaciers. In all cases, these aerofoil features are isolated from the main region of surging, are oriented at roughly the same angles in the parameter space, have long-axis lengths that scale nonlinearly with *ϵ*_*e*_/*ϵ*_*p*_, and have positions that shift towards higher *t*_*h*_ and smaller *b* as *ϵ*_*e*_/*ϵ*_*p*_ increases. The boundaries of these features are diffuse in the direction of smaller *t*_*h*_ and *b* but feature sharp transitions in both maximum and final slip rates at higher *t*_*h*_ and *b* values. Second, the boundary separating the surging region from the non-surging and abandoned surge regions is sharp, rather than diffuse, suggesting the existence of a supercritical Hopf bifurcation at the (approximately) linear boundary between surging and non-surging in the *t*_*h*_–*b* parameter space. As expounded on in the Discussion, this sharp boundary and possible bifurcation illuminates some potential mechanisms that cause surging to switch on and off over longer (multi-centennial) time scales in a given glacier system, and for surging glaciers to be relatively rare and geographically clustered. We reserve for future work detailed exploration of bifurcations in the system.

To better understand the features in figure 5, we explore the dynamics in figure 6, which shows that small variations in *b* for fixed values of *t*_*h*_ and *ϵ*_*e*_/*ϵ*_*p*_ lead to a range of responses. The parameter values represented in figure 6 are shown with corresponding colours in figure 5. In order of decreasing *b*, we observe surging following the perturbation (blue line; *b* = 0.03), abandoned surging (orange line; *b* = 0.028), an abandoned surge followed by a surge at longer time scales (red line; *b* = 0.026) and slight dynamical variations (green and olive lines; *b* ≤ 0.024). These transitions in dynamical behaviour as a function of decreasing *b* can be understood in the context of changes in *μ*, the internal friction coefficient of the till. The sensitivity of *μ* to changes in state increases with *b*, allowing for greater and more rapid reductions in the friction coefficient—and, by extension, the shear strength of the till, *τ*_*t*_ (lowest panel of figure 6)—at higher *b* values. Thus, higher *b* values lead to unstable acceleration immediately following the perturbation by allowing dynamic glacier thinning to drive a net reduction in the effective pressure, further decreasing the shear strength of the till. Slightly smaller *b* values in the abandoned surge region result in slightly smaller changes in *μ*, which creates a situation that is unfavourable to surging because the acceleration in basal slip rate is sufficiently fast to drive till evolution but not significant dynamic thinning of the glacier. As a result, the initial acceleration is facilitated by reductions in both the effective pressure and internal friction coefficient, but decreases in pore water pressure eventually outpace reductions in overburden pressure, resulting in a net increase in effective pressure (and *τ*_*t*_) and ultimate stagnation of basal slip. Finally, a delayed surge manifests at median *b* values (*b* = 0.026 for *t*_*h*_ = 2600 days; red line in figure 6) because of trade-offs in basal slip acceleration, till dilation and evolution of the internal friction coefficient. In this case, small initial decreases in *μ* driven by state evolution allow for basal slip acceleration, which drives the till towards steady state and ultimately increases state *θ* beyond the initial steady-state value as the glacier slows. Since basal slip does not stagnate as it did in the previously discussed case, the till continues to evolve, eventually leading to compaction and commensurate increase in pore water pressure. This increase in pore water pressure drives a reduction in effective pressure that leads to glacier acceleration, which eventually becomes self-sustaining as the glacier thins and effective pressure drops.

We find good agreement between our model behaviour and observations of surge motion in two natural glaciers (figure 7). Our model reproduces both the timing and order of magnitude of the speed-up with *b* = 0.03 and *t*_*h*_ = 3000 days and other parameters corresponding to values used in figures 3 and 4. Note that our focus in this study has been on the incipient acceleration phase of the surges. Simplifications in the model, namely the lack of an evolving subglacial hydrological system and consideration of extensional stresses in the momentum balance, prevent the model from decelerating [10]. The agreement between our model and these data, however, is encouraging as it suggests that the dilation and glacier-thinning time scales we consider in our model may work in concert to trigger glacier surges.

## 4. Discussion

At this point, we have derived and explored the behaviour of a fundamentally new dynamical model of incipient surge motion that considers the mechanics of subglacial till and ice flow. Few comparable models exist in the literature, thus we endeavour to develop the simplest model capable of capturing the salient physical processes of ice slipping due to deformation of beds composed of water-saturated till. As detailed later in this section, natural glacier systems will, of course, be more complex than our model. Nevertheless, our model evinces rich dynamical behaviours consistent with observations, suggesting that our model strikes an appropriate balance between capturing the salient physical processes while remaining simple enough to allow for physical insight.

### (a) Mechanics of incipient surge motion

Rich dynamical behaviour in our model is driven by the interactions of the three factors that define the shear strength of the till *τ*_*t*_ = (*p*_*i*_ − *p*_*w*_)*μ*: the overburden pressure *p*_*i*_ = *ρ*_*i*_ *g h*, pore water pressure *p*_*w*_, and the rate-and-state-dependent internal friction coefficient *μ* = *μ*(*u*_*b*_, *θ*). To understand surge behaviour in glaciers with till-covered beds, it is important to recognize that pore water pressure tends to decrease as a result of dilation, which strengthens till and resists surge motion, while the internal friction coefficient can increase or decrease, often by small amounts. Rate-weakening internal friction (*a* − *b* < 0) can help to facilitate surges but is not a necessary condition as surges are possible with rate-strengthening friction coefficients (*a* − *b* > 0) under conditions that allow for reductions in effective pressure (figure 5).

The key process governing incipient surge motion is suction caused by till dilation in relatively impermeable till. In this case, pore water pressure decreases in response to shear-driven dilation, and the drop in pore water pressure diminishes the ability of till to evolve to a new steady state. If hydraulic permeability is sufficiently low (i.e. if the diffusion time of the deforming till layer *t*_*h*_ is sufficiently long), slowing of state evolution allows the glacier to accelerate for longer periods of time. This longer acceleration phase gives the glacier time to thin dynamically, which reduces the overburden pressure (*p*_*i*_). In the region of the parameter space shown in figure 5, the reduction in overburden pressure outpaces drops in pore water pressure (*p*_*w*_), leading to a net reduction in the effective pressure (*N* = *p*_*i*_ − *p*_*w*_) and thereby the shear strength of till (*τ*_*t*_ = *μN*). From equations (2.24) and (3.4), we can see that the rate of change in driving stress is ${\dot{\tau }}_{d}\approx 2{\dot{p}}_{i}\alpha$, indicating that driving stress evolves at least an order of magnitude more slowly than changes in overburden owing to the shallow slopes of glaciers (*α* ≪ 1). As a result, reductions in overburden pressure facilitate sustained excess driving stress (*τ*_*d*_ > *τ*_*b*_), the key ingredient for sustained incipient surge motion. It is necessary, then, that the initial acceleration be large enough and last for long enough to generate sufficient dynamical thinning of the glacier.

### (b) Implications of surge mechanics

The need for dynamic thinning to sustain surge motion gives two necessary conditions for glacier surging: till must have sufficiently low hydraulic permeability to allow for incipient surge motion to be maintained over a long enough period of time, and the velocity during the nascent surge must exceed the balance velocity to allow for dynamical thinning. The latter condition implies a third necessary condition: shear strength of the till must be less than the balance driving stress, defined as the driving stress at which the balance velocity is achieved through internal deformation of the ice column. Consequently, yielding of the till must occur at glacier velocities slower than the balance velocity to allow for continual shear loading of the till.

In the accumulation zones of surging glaciers, flow speeds must be slower than the balance velocity to build an ever-thickening reservoir of ice [15]. This condition must persist throughout the quiescent phase because, once the flow speed reaches the balance velocity, there would be no way to further increase driving stress and load the bed as ice mass would be evacuated by flow accommodated through vertical shearing of the ice column. In other words, mass balance along with the geometric and rheological properties of surge-type glaciers allow them to build a reservoir that exerts a driving stress equal to bed failure strength before flow rates reach the balance velocity. To illustrate this point, consider that the maximum shear stress a glacier can apply to its bed is given by the gravitational driving stress when the surface velocity of the ice equals the balance velocity and basal slip rate is negligible (*τ*_*b*_ ≈ *τ*_*d*_). Surface velocity due solely to vertical shearing within the ice column *u*_*v*_ is given by assuming that stress increases linearly with depth, that ice rheology is constant with depth and that ice flow is parallel to the ice surface, yielding

4.1 $$u_{v}=\frac{2Ah\tau_{d}^{n}}{n+1},$$

where *A* is the prefactor and *n* is the stress exponent in the constitutive relation for ice (equation (2.25)). The driving stress at which *u*_*v*_ matches the balance velocity $u_{*}=\dot{M}/(\zeta \alpha )=\dot{M}(n+2)/[\alpha (n+1)]$ (cf. equations (3.2) and (3.3)) is then

4.2*a,b* $$\tau_{d*}={\left(\frac{\dot{M}(n+2)}{2Ah\alpha }\right)}^{1/n}={\left(\frac{\rho_{i}g\dot{M}(n+2)}{2A}\right)}^{1/(n+1)},$$

where equation (4.2*b*) comes from recognizing that *α* = *τ*_d*_/(*ρ*_*i*_ *g h*). The variables $\dot{M}$, *A* and, to a lesser extent, *ρ*_*i*_ and *n* are governed by local climate [94]. Although mass density cannot vary more than 25% and *n* should be approximately 3, $\dot{M}$ and *A* can vary independently by orders of magnitude. Thus, the balance driving stress, *τ*_d*_, for an idealized glacier is determined primarily by $\dot{M}/A$, the ratio of mass balance, $\dot{M}$, to the rate factor, *A*, the latter of which depends on ice temperature and interstitial meltwater content, along with crystallographic fabric [104].

Equation (4.2) underpins a necessary condition for surging: at a minimum, surging glaciers must have a climate, and geometry, that allows for sufficiently high balance driving stresses (*τ*_d*_)—a combination of high, positive mass balance and stiff ice (small *A*)—to overcome the strength of their beds. As a result, the geographic distribution of surge-type glaciers will reflect areas that combine sufficiently high rates of snowfall, relatively low summertime melt at the surface and cold, stiff ice with beds that have yield stresses below the respective *τ*_d*_ but are strong enough to allow the glacier to develop driving stresses that allow for order-of-magnitude increases in ice flow during the surge. To get a rough estimate for the pre-surge driving stress needed to produce a given speed-up, let us assume that the pre-surge surface velocity, $u_{s_{\text{pre}}}$, in the region where a surge begins is primarily due to viscous deformation in the ice column (given by equation (4.1)) and that surface velocity at peak surge speeds, $u_{s_{\text{surge}}}$, is due primarily to basal slip (given by equation (2.26)). Taking the ratio $u_{s_{\text{pre}}}/u_{s_{\text{surge}}}$ and rearranging the terms (recalling that *h* ≪ *w*) gives

4.3 $$\tau_{d_{\text{pre}}}\approx \tau_{t_{\text{surge}}}\left[1+{\left(\frac{u_{s_{\text{surge}}}}{u_{s_{\text{pre}}}}\frac{h_{\text{surge}}^{n}h_{\text{pre}}}{w^{n+1}}\right)}^{1/n}\right],$$

where $\tau_{t_{\text{surge}}}$ is the shear strength of the till when the glacier is flowing at peak surge speed. Combining equation (4.3) with the balance velocity explicitly gives the necessary condition

4.4 $$\tau_{d_{\text{pre}}}<\tau_{d*},$$

which to a good approximation is simply ${\bar{\tau }}_{t}<\tau_{d*}$, where ${\bar{\tau }}_{t}$ is the long-term average shear strength of the till in the region where surges nucleate. The range of reasonable values on *ρ*_*i*_ *g* is small, so, to a good approximation, whether a glacier meets the condition in equation (4.4) is determined primarily by mass balance, ice rheology, bed strength and cross-sectional aspect ratio (*h*/*w*).

**Figure 8.**
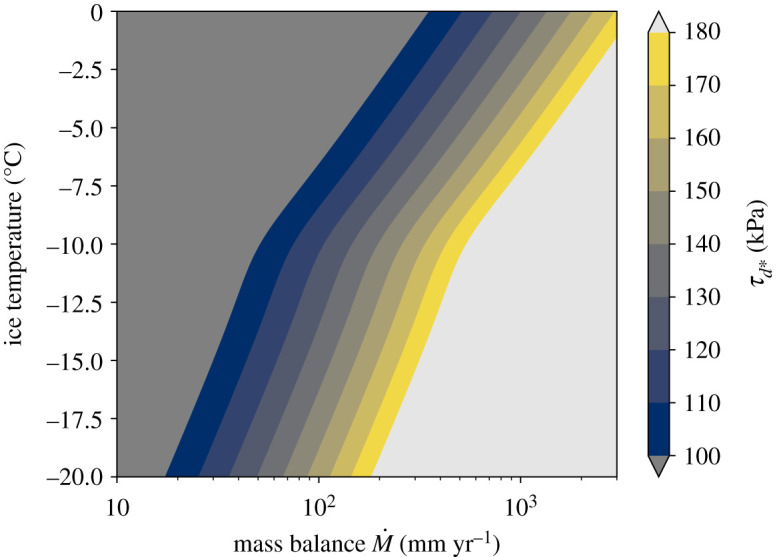
Balance driving stress *τ*_d*_ (equation (4.2)) as a function of surface mass balance ($\dot{M}$) and ice temperature. The rate factor is taken to depend on ice temperature *T* according to the Arrhenius relation $A=A_{*}\exp\left\{-Q_{c}(T^{-1}-T_{*}^{-1})/R\right\}$, where $T_{*}=-10^{\circ }\text{C}$, $A_{*}=3.5\times 10^{-25}\;{\text{Pa}}^{-3}\;{\text{s}}^{-1}$, *Q*_*c*_ is the activation energy that increases from 60 kJ mol^−1^ for $T\leq T_{*}$ to 115 kJ/mol for $T_{*}<T\leq 0^{\circ }\text{C}$, and *R* = 8.314 J/(K · mol) is the ideal gas constant [94]. The colourmap is defined to capture the range of driving stresses typically found on Earth [42]. (Online version in colour.)

The conditions defined by equations (4.2)–(4.4) yield surge conditions discussed in previous observational studies. The dependence on mass balance is consistent with observations that have shown cumulative quiescent-phase mass balance to be a reliable predictor of surging on Variegated Glacier, Alaska [105,106]. The temperature-dependent ice rheology broadly agrees with the climatic trends reported in [12] for surge-type glaciers (figure 8). In this framework, warmer climate (and ice temperatures) require higher values of surface mass balance to satisfy the condition that the bed yields before the driving stress becomes high enough to cause the glacier to flow at the balance velocity through internal deformation within the ice.

Further insight into the spatial distribution and longer term evolution of surge-type glaciers can be gleaned from the boundaries between surge-type and non-surge-type glaciers illuminated in the permeability versus evolution effect parameter space (figure 5). The sharp, diagonal boundary between surging on non-surge behaviour suggests the existence of a bifurcation in the system and lies at values that are likely to be relatively rare in nature and closely linked to local lithology and degree of weathering. In particular, our model suggests that values of hydraulic diffusivity for till in surge-type glaciers fall in the lower range of observed values (∼ 10^−9^ m^2^ s^−1^) for the range of *b* values explored in this study. Such low hydraulic diffusivities are consistent with canonical values of permeability expected for fine-grain sediments and loams [50,94]. The need for such low values of hydraulic permeability and fine-grained sediments suggests a potential role for comminution and sediment transport in activating and deactivating surging over millennial time scales, though future work is needed to elucidate these connections.

The governing role of till dilation and evolving pore water pressure in our model points to further methods for testing the model in nature. In addition to the comparisons with data similar to those given in this study (namely figure 7 and the preceding discussion of geographical distribution of surge-type glaciers), we propose that passive seismic data collected during the incipient surge phase would provide valuable insight into the salient processes and could be used to test our model. Passive seismic data are routinely used to estimate the seismic moment from which estimates of the bulk shear modulus can be gleaned. The shear modulus is sensitive to both the porosity and pore water pressure, and so can be used as a means to observe till dilation and variations in pore water pressure.

### (c) Model limitations and future development

Our goal with this work is to better understand basal mechanics by developing a model for incipient surge motion in glaciers with till-covered beds. We do not attempt to capture all of the processes that may be important in initiating and sustaining glacier surges. As a result, our model has some limitations that provide avenues for future work.

A notable limitation is the lack of explicit treatment for evolution of the subglacial hydrological system during any stage of the surge or the quiescent phase. The influence of basal hydrological characteristics is manifested in the model through the system water pressure $p_{w_{r}}$, but we implicitly treat this water pressure as passive in the model development. A fully passive basal hydrological system is unlikely given the rapid, extreme changes in glacier dynamics that define a surge. During surges, significant volumes of till are displaced, filling most existing cavities, basal crevasses or channels that constitute the contemporaneous hydrological system [17]. This lack of explicit treatment for changes in $p_{w_{r}}$ owing to till displacement leaves open the possibility that increases in basal water pressure caused by changes in the basal hydrological system can cause surges. What we have provided in this study are proposed mechanisms of incipient surge motion in glaciers with deformable beds that are not dependent on changes in the basal hydrological system. The existence of such a mechanism, which works equally well for temperate and polythermal glaciers, and observations of surges beginning in times of the year when there is little or no additional surface meltwater available to pressurize a basal hydrological system (e.g. during winter) support the hypothesis that it is the incipient surge motion that diminishes the efficiency of any extant hydrological system rather than changes in the hydrological system that lead to surges [10].

We do not explicitly consider enhanced melting of basal ice through frictional heating or viscous dissipation, the former being a key mechanism in some existing surge models [10]. The reasons for this exclusion are twofold. First is the model set-up. We focus on instabilities that may arise from till mechanics and assume that rapid flow is due to basal slip, and basal slip is due entirely to deformation of the till. Thus the ice is in stationary contact with the top layer of the till and vertical shearing within the ice column is negligible. The latter case minimizes melting of basal ice through viscous dissipation. For frictional heating, the rate of melt scales with the product of rate of sliding along the ice–bed interface (i.e. the velocity of the ice relative to the top of the till) and the drag at the interface. As a result, there is no frictional heating in our model because we do not allow sliding at the ice–till interface. The addition of sliding at the ice–till interface is an appealing avenue for future research as the effective rheology of this interface and the resultant heating are non-trivial given that deformation of the bed is through cataclastic flow, facilitated by boundary sliding and rolling of sediment grains, and should facilitate comminution. The second reason we exclude slip-induced melting is that melting only influences ice dynamics through changes in basal and pore water pressure. Without a reliable model for subglacial hydrology on deforming sediment, there is no way to effectively link basal melt rate and water pressure. However, we reiterate that our model produces surge-like behaviour without representing frictional heating or evolving subglacial hydrology, suggesting that these mechanisms are not necessary for surge-like behaviour. The existence of unstable sliding phenomena, such as earthquakes and landslides, that do not rely on frictional heating or evolving hydrology at the slip interface supports our supposition that these mechanisms are not general requirements for surges, though they undoubtedly influence glacier surge dynamics [10]. The only general requirement for unstable acceleration is the existence of mechanisms that allow for the sustainment of excess driving forces relative to resistive forces, which our model achieves through reductions in overburden (and, thereby, effective) pressure.

Finally, our model does not capture the down-glacier propagation of mechanical, kinematic or basal water pressure waves [21,107,108]. This limitation arises from the fact that our model is essentially one-dimensional (in the vertical), meaning that we neglect the gradient of extensional (along-flow normal) stresses and strain rates (equations (2.26) and (3.3)) along with horizontal gradients in water pressure. During the quiescent phase, neglecting extensional stresses is reasonable in the upper accumulation zone where surges are prone to begin. Here, surface velocities tend to be slow and relatively consistent over large spatial scales, meaning that along-flow strain rates are small relative to the effective strain rate; since ice is a viscous fluid, low strain rates mean low stresses. During the surge, the surface velocities are high, with the exception of the period when surge waves are present, and velocities can be expected to have small spatial gradients [6,29]. A more complete model of glacier surges would include more terms of the stress divergence such that it could account for the propagation of surge motion through the glacier. This more complete model would be useful for further investigating the influence of glacier length on surge behaviour [10]. However, we consider our box-model analysis to be a prerequisite to more complicated flowline and three-dimensional studies, which we reserve for future work.

## 5. Summary and conclusion

In this paper, we develop a new model of incipient surge motion in glaciers with till-covered beds. Incipient surge motion in our model occurs in the absence of enhanced water flux to the bed, changes to the basal hydrological system, frictional heating due to slip at the ice–bed interface and freeze–thaw cycles in till. Our model is based on granular mechanics of the till and focuses on processes that can lead to unstable acceleration in glaciers with deformable beds. Our model is unique among existing surge models in that it accounts for till porosity and pore water pressure, and represents the evolution of internal friction, porosity and pore water pressure within the deforming till layer as functions of the rate and history of shearing within the deforming till layer. This combination of mechanisms allows for exploration of the rich dynamics that arise from changes in the three factors that govern the shear strength of till: ice overburden pressure, pore water pressure and the internal friction coefficient. To represent these factors, we adopt the phenomenological rate-and-state model commonly used in studies of slip on tectonic faults. We link the state variable, which encodes the history of basal slip, to till porosity and derive a model in which pore water pressure evolves as a result of changes in porosity and transport of pore water (i.e. Darcy flow) into and out of the deforming till layer.

We find that till dilation, and more specifically suction caused by the reduction of pore water pressure in response to dilation, is a fundamental control on incipient surge motion. This control arises from the need for dynamic thinning of the glacier to sustain surge motion by reducing the effective pressure at the bed. Glacier thinning is necessary because, following a perturbation, till tends toward a new steady state while flow of water into and out of the deforming layer acts to equalize pore water pressure between the underlying static till layer, the deforming till layer and the subglacial hydrological system. As a result, the shear strength of the bed tends to a new steady state, leading to stable acceleration, unless the glacier thins. If the permeability of the till is sufficiently low, the evolution of the till to a new steady state is slow enough to allow accelerated surge motion to thin the glacier, as long as flow speeds during the nascent surge exceed the balance velocity of the glacier. Thinning of the glacier allows for unstable acceleration of the glacier owing to reductions in effective pressure, and, consequently, shear strength of the till, leading to order-of-magnitude increases in flow velocity that characterize surges and are consistent with observations of glacier acceleration during surges.

The hydromechanical properties of till, namely the need for low till permeability, required to induce rapid glacier thinning and surge motion give rise to restrictive conditions for glacier surges and rich dynamics. The necessary conditions for surging illuminated by our model are low hydraulic permeability in the deforming till layer, surge velocities that exceed the balance velocity and maximum shear strength of till that is less than the driving stress needed to achieve the balance velocity through vertical shearing in the ice column. These conditions are consistent with the rarity of surge-type glaciers; the geographical and climatic distribution and clustering of surge-type glaciers; and millennial time-scale evolution of surge behaviour. Furthermore, the rich dynamics produced by our model allow for abandoned surges along with a spectrum of surge-like behaviours that are consistent with kinematic observations of natural glaciers but are lacking in existing surge models.

Our model is necessarily simplified but contains important new physical processes—namely, till mechanics—that have been neglected in virtually all previous studies of glacier surges. To focus on the complex processes of water-saturated till, we deliberately ignore other processes that may be essential for a complete understanding of surge dynamics. Most notably, we neglect extensional stresses and vertical shearing in the ice column, and we treat the subglacial hydrological system as static. As a result, our model only captures the incipient surge phase and not slowdowns that terminate surges. We derive our model such that the inclusion of a dynamic subglacial hydrological system should be a relatively straightforward addition, and extension and vertical shear stresses can be included with the application of a more sophisticated flow model that accounts for more terms of the stress divergence in the momentum equations. These avenues provide numerous opportunities for future exploration of surge dynamics.

## Supplementary Material

Supplemental Material

## Data accessibility

No new data are presented in this study. Source code for the numerical simulations is available at github.com/bminchew/glacier_surging1.git.
