# Supplementary material for: Dilation of subglacial sediment governs incipient surge motion in glaciers with deformable beds
Source: Proc Math Phys Eng Sci. 2020 Jun 3;476(2238):20200033. doi: 10.1098/rspa.2020.0033 (PMC7428031; doi:10.1098/rspa.2020.0033)
Supplement: Supplemental Material [file rspa20200033supp1.pdf]

Supplemental Material for "Dilation of subglacial sediment governs incipient surge motion in glaciers with deformable beds"

Brent M. Minchew[1] and Colin R. Meyer[2]
[1]Department of Earth, Atmospheric and Planetary Sciences, Massachusetts Institute of Technology, Cambridge, MA, USA
[2]Thayer School of Engineering, Dartmouth College, Hanover, NH, USA
Corresponding author: B. M. Minchew, minchew@mit.edu

Notation

Variables	Descriptions	Units
a	direct (velocity) effect on coefficient of internal friction	-
b	evolution effect on coefficient of internal friction	-
A	rate factor in constitutive relation for ice	Pa^{-n} s^{-1}
d_c	characteristic slip displacement	m
μ	coefficient of internal friction of till	-
μ_n	nominal coefficient of internal friction	-
g	gravitational acceleration	m s^{-2}
h	ice thickness	m
h_s	thickness of deformable till layer	m
ℓ	glacier length	m
m_w	water mass per unit volume of till	kg m^{-3}
$\dot{M}$	glacier surface mass balance	m s^{-1}
N	effective pressure at the glacier bed ($N = p_i - p_w$)	Pa
n	stress exponent in constitutive relation for ice	-
p_i	ice overburden pressure ($p_i = \rho_i g h$)	Pa
p_w	pore water pressure in deformable till layer	Pa
p_{w_∞}	pore water pressure in non-deforming substrate	Pa
p_{w_r}	water pressure in subglacial hydrological system	Pa
q_w	water flux in deformable till layer	kg m^{-2} s^{-1}
t_h	hydraulic diffusion timescale of deformable till layer	s
$\bar{u}$	depth-averaged speed of glacier	m s^{-1}
u_b	basal slip rate	m s^{-1}
u_{b_n}	nominal basal slip rate	m s^{-1}
u_s	surface speed of glacier	m s^{-1}
u_{s*}	balance surface speed	m s^{-1}
w	glacier half-width	m
α	ice surface slope	-
β	till compressibility	Pa^{-1}
γ_h	till permeability	m^2
ϵ_e	elastic compressibility coefficient	-
ϵ_p	plastic dilatancy coefficient	-

$\dot{\varepsilon}_{ij}$	strain rate tensor	s^{-1}
$\dot{\varepsilon}_e$	effective strain rate ($\dot{\varepsilon}_e = \sqrt{\dot{\varepsilon}_{ij}\dot{\varepsilon}_{ij}/2}$)	s^{-1}
ζ	ratio of depth-averaged velocity to surface velocity	-
κ_h	hydraulic diffusivity of till	$m^2\ s^{-1}$
θ	state of deformable till	s
η_w	dynamic viscosity of water	Pa·s
ρ_i	mass density of ice	kg m^{-3}
ρ_w	mass density of water	kg m^{-3}
τ_{ij}	deviatoric stress tensor	Pa
τ_b	basal drag	Pa
τ_d	gravitational driving stress	Pa
τ_{d*}	balance driving stress	Pa
τ_e	effective deviatoric stress ($\tau_e = \sqrt{\tau_{ij}\tau_{ij}/2}$)	Pa
τ_t	till shear strength	Pa
ϕ	till porosity	-
ϕ_p	plastic component of till porosity	-
ψ	hydraulic transmittance	-
